# Development and Evaluation of Nanoemulsifying Preconcentrate of Curcumin for Colon Delivery

**DOI:** 10.1155/2015/541510

**Published:** 2015-03-11

**Authors:** Jyoti Wadhwa, Abhay Asthana, Gyati Shilakari, Arun Kumar Chopra, Ranjit Singh

**Affiliations:** ^1^M. M. College of Pharmacy, M. M. University, Mullana, Ambala 133207, India; ^2^International Institute of Pharmaceutical Sciences, Jhundpur, Sonepat 131023, India; ^3^Government College of Pharmacy, Rohru, Shimla 171207, India

## Abstract

The present study aimed to develop and optimize a nanoemulsifying preconcentrate formulation of curcumin with good emulsification ability and optimal globule size, for controlled targeting in colon. Content of formulation variables, namely, *X*_1_ (Peceol), *X*_2_ (Cremophor-EL), and *X*_3_ (Transcutol HP), were optimized by Box-Behnken design of experiments for its impact on mean globule size (*Y*_1_), emulsification time (*Y*_2_), and time required for drug release (85%) in phosphate buffer (pH 7.2), *t*_85%_ (*Y*_3_). Transmission electron micrographs confirmed that there is no coalescence among globules, with size range concordant with the globule size analysis by dynamic light scattering technique (100 nm). 3D plots indicated that concentration of formulation ingredients significantly influences the formulation properties (globule size, emulsification time, and drug release). *In vitro* release profile (in phosphate buffer; pH 7.2) represents the fact that more than 50% of the drug was released within initial 15 min whereas *in vivo* release showed limited systemic absorption (*C*_max_ 200 ng/mL) of curcumin. Stability study ensures the protection of drug in alkaline media which may further confirm the localised delivery of drug to colonic region. Study demonstrated that the nanoemulsifying preconcentrate can be a promising system for the colon specific delivery of curcumin to treat local pathologies.

## 1. Introduction

Majority of upcoming drugs, as well as those currently in development, are highly lipophilic in nature and thus utilization of novel drug delivery approach in formulation of drug products is desirable [1]. Various formulation strategies are currently being employed to tackle drug delivery challenges of such critical molecules, either by predissolving them in a suitable solvent and subsequently filling the formulation into capsules [2] or by formulating as solid solution using water-soluble polymers [3]. These approaches can probably resolve the issue related to initial dissolution of drug molecules in aqueous environment within the GI tract to certain extent. However, major limitations like drug precipitation during dispersion of formulation in the GI tract and drug crystallization in the polymer matrix remain unresolved. Therefore, in case of such formulations, the assessment of physical stability using techniques such as differential scanning calorimetry and X-ray crystallography is necessary.

One of the major advancements in the areas of drug delivery was the recognition of benefits of formulating highly lipophilic actives as lipoidal formulations [4]. Lipids are perhaps one of the most versatile excipient classes currently available and provide the formulator potential option to improve and control the absorption of lipophilic drugs, where typical formulation approaches failed or when the drug itself is oil (i.e., dronabinol, ethyl icosapentate). Moreover, with such formulations, there is lower potential for precipitation of lipophilic drug molecules during dilution in the GI tract, as partitioning kinetics will favor the drug to remain in the lipid droplets [5]. Lipoidal formulation is an isotropic mixture of oil, surfactant, cosurfactants, and drug and can form nanoemulsions under gentle agitation [6], which can be further transformed into solid form like powder and tablet or capsule dosage form by adsorption on solid carrier [7, 8].

Curcumin (CUR), a naturally occurring polyphenolic compound, is a potential adjuvant to anticancer chemotherapy. Recent studies have shown that CUR interferes with the propagation of colon cancer [9]. Unlike other anticancer drugs that weaken the immune system, CUR acts as an “immunorestorer” [10]. CUR, however, poses a challenge during formulation development, owing to poor water solubility and rapid intestinal metabolism, thus limiting the industrial utility of CUR. In order to address such limitations, various delivery systems have been investigated, including cyclodextrin complexation [11, 12], solid dispersion [13, 14], liposomes [15], phospholipid complexes [16], solid lipid nanoparticles [17], polymeric nanoparticles [18], nanocrystals [19], and nanoemulsions formulation [20]. However, few of them have characteristic shortcomings, including poor physical stability, drug leakage, and potential toxicity of excipients. On the other hand, nanoemulsifying preconcentrate formulations (NP) represent a novel delivery system that pools the benefits of an emulsion without stability issues while providing the biological compatibility of lipid carriers [21]. Additionally, it can be filled in capsules due to their anhydrous nature, providing a convenient and patient compliant approach [22]. Probably the best known example is Sandimmune-Neoral (microemulsion preconcentrate of cyclosporine), which reduces the highly variable pharmacokinetic profile of cyclosporine [23]. Such formulations have also been widely used to improve oral bioavailability of drugs, particularly those belonging to BCS class II and IV drugs [24]. Mechanisms of improvement include improved solubility, changing intestinal permeability, and interfering with enzymes and transporter activity via bioactive lipid excipients and surfactants [25–27].

Owing to poor solubility and extensive presystemic clearance of CUR parallel to the reported advantages of lipoidal formulation, the present study was aimed at optimizing CUR loaded nanoemulsifying preconcentrate formulation with good emulsification ability and optimal globule size for controlled targeting in colon. Box-Behnken design of experiments was applied to investigate the influence of oil percentage and surfactant to cosurfactant (*S*_mix_) ratio on the formulation variables (globule size, *t*_85%_ and emulsification time). This study could help in developing a novel optimal emulsifying preconcentrate for delivery of CUR.

## 2. Materials and Methods

### 2.1. Materials

Curcumin (CUR) was obtained as* gratis* sample from Himedia, Mumbai, India. Transcutol HP (T-HP), Lauroglycol FCC (LFCC), Peceol, Capryol 90, and Capryol PGMC were obtained from Gattefosse Pvt. Ltd (Mumbai, India). Cremophor-EL (C-EL) and edible oils (isopropyl myristate, ethyl oleate, castor oil, arachis oil, lemon oil, oleic acid, apricot oil, olive oil, corn oil, Captex 200, and soybean oil) were purchased from Himedia (Mumbai, India). Tween 80, Span 80, Tween 20, and Aerosil 200 were purchased from Merck (Mumbai, India). Capsule shells were purchased from Torpac Inc. (USA). Eudragit S100 was kindly gifted by Evonik India Pvt. Ltd. (Mumbai, India). All other chemicals and reagents used in the study were of analytical grade.

### 2.2. HPLC Analysis of CUR

Reverse phase HPLC system (LC-2010 CHT; Shimadzu, Japan) comprising Phenomenex C18 column (250 mm × 4.6 mm) was used for analysis of CUR. Mobile phase was an isocratic mixture of acetonitrile: HPLC water (57 : 43% v/v), at pH 3.3 maintained using citric acid. Elution was carried out at a flow rate of 1.0 mL/min at room temperature (37°C), With the UV-Vis detection wavelength of 425 nm. Method was validated using various parameters such as accuracy, precision, linearity, limit of detection, and limit of quantification. Summary of the validation parameters is reported in Table 1.

### 2.3. Initial Screening of Excipients

#### 2.3.1. Solubility Study

Solubility of CUR was determined in different vehicles, that is, oils (isopropyl myristate, ethyl oleate, castor oil, arachis oil, lemon oil, oleic acid, apricot oil, Peceol, olive oil, corn oil, soybean oil, Labrafac, and Captex 200), surfactants (Tween 20, Tween 80, Span 80, Cremophor-EL, and Lauroglycol FCC), and cosurfactant, using the saturated shake flask method. Excess CUR was suspended in the respective vehicles in screw capped glass vials. Mixture was vortexed (Remi motors Pvt. Ltd., India), followed by sonication (5 min), to ensure uniform mixing. Mixtures were subjected to uniform shaking at 37°C for 24 h in shaker water bath (Acumax India Pvt. Ltd., New Delhi, India) set at 100 rpm and allowed to stand for 48 h to attain equilibrium. After 72 h, mixtures were centrifuged at 1300 ×g for 10 min, followed by filtration through a 0.45 *μ*m Millipore membrane filter. Filtrate was diluted with methanol and quantified using HPLC.

#### 2.3.2. Ternary Phase Diagrams

Based on CUR solubility in various vehicles, ternary phase diagram was developed for selected oil, surfactant, and cosurfactant mixture, using the aqueous titration method. Three variables (factors) were used, that is, oil, water, and mixture of surfactant and cosurfactant (*S*_mix_) in specific ratio (i.e., 1 : 2, 2 : 1, and 3 : 1). Titration with aqueous phase was carried out at each weight ratio of oil to *S*_mix_, within the range of 1 : 9 to 9 : 1. Visual observation was performed to check formation of transparent and easily flowable oil/water nanoemulsion. Physical state of the nanoemulsion was marked on a pseudo three-component phase diagram, constructed using PCP Disso Ver. 3.0 software. One of the axes was the aqueous phase, the second was oil, and the third was the mixture of surfactant and cosurfactant at fixed weight ratios (*S*_mix_ 1 : 2, 2 : 1, and 3 : 1). Phase boundary was determined by observing the change in sample appearance from transparent to turbid phase transition [28].

#### 2.3.3. Preparation of Liquid Nanoemulsifying Preconcentrate (CUR-LNP)

Based on the formation of maximal nanoemulsion region in the ternary phase diagram, a three-factor, *X*_1_ (Peceol), *X*_2_ (Cremophor-EL), and *X*_3_ (Transcutol HP), three-level (−1, 0, and +1) design was applied for the optimization procedure using Design-Expert 8.0.7.1 software. A set of seventeen experimental runs comprising independent factors and the dependent variables (responses) were studied as per the experimental design matrix (Table 2). The quadratic model generated by the design has the following form:

(1) Y=b0+b1X1+b2X2+b3X3+b12X1X2+b13X1X3+b23X2X3+b11X12+b22X22+b33X32.

The above equation comprises coefficient of intercept, first-order main effect (*X*_1_, *X*_2_, and *X*_3_), interaction terms (*X*_1_ *X*_2_, *X*_1_ *X*_3_, and *X*_2_ *X*_3_), and high order effects (*X*_1_^2^, *X*_2_^2^, and *X*_3_^2^), where *Y* is the measured response; response variables selected for the optimization purpose were mean globule size (*Z*-avg) in phosphate buffer (pH 7.2) (*Y*_1_), emulsification time (*Y*_2_), and the time taken for 85% release of the drug, *t*_85%_ (*Y*_3_).

CUR (50 mg) was dissolved in oil using vortexer (Remi Motors Ltd., Mumbai, India). Oil phase containing CUR was transferred into the surfactant and cosurfactant mixture, under continuous mixing at 50°C until CUR was completely dissolved. The generated isotropic mixture was stored at ambient temperature, until further use. Formulations were further evaluated for emulsification time, droplet size, and the* in vitro* release rate.

### 2.4. Evaluation of Liquid Nanoemulsifying Preconcentrate (CUR-LNP)

#### 2.4.1. Emulsification Time

Time required for emulsification of CUR-LNP formulation was determined by the method described by Khoo et al. [29]. Each formulation was added dropwise to phosphate buffer (pH 7.2) and agitated at 50 rpm. Formation of emulsion was observed visually and the time taken was recorded. All measurements were performed in triplicate.

#### 2.4.2. Globule Size Determination

Globule size of CUR-LNP formulation was determined using dynamic light scattering (DLS) technique, with Zetasizer (Nanosizer) Nano S90 (Malvern Instruments, UK). LNP formulation containing equivalent amount of CUR (10 mg) was diluted using 100 mL phosphate buffer (pH 7.2) [30]. Upon dispersion, globule size was determined. All measurements were performed in triplicate.

#### 2.4.3. *In Vitro* Dissolution Studies

*In vitro* drug release study was performed in USP type II (Paddle type) dissolution apparatus, maintained at 37 ± 0.5°C. Formulation equivalent to 100 mg of CUR was filled in hard gelatin capsules shells and sealed. sealed capsules were further coated with Eudragit S100 (6% w/w). Drug release from capsule filled with CUR-LNP was studied for 2 h in simulated gastric fluid (pH 1.2), phthalate buffer (pH 4.5) for 2 h, phosphate buffer (pH 6.8) for 1.5 h, and phosphate buffer (pH 7.2) for 1 h. Aliquot of 5 mL dissolution medium was withdrawn at predetermined time intervals and filtered through 0.45 *μ*m membrane filter. Samples were analysed using HPLC.

#### 2.4.4. Preparation of Solid Nanoemulsifying Preconcentrate (CUR-SNP)

CUR-SNP formulation was prepared by adsorption of LNP formulation onto Aerosil 200 as the solid carrier. Accurately weighed, optimized LNP formulation (P3), containing 100 mg equivalent weight of CUR, was adsorbed onto Aerosil 200 in the ratio of 1 : 1, 1 : 5, and 1 : 10 (LNP: Aerosil 200). Adsorbed solid particles (SNP) were sieved through mesh number 120. SNP powder formulation was stored in a desiccator at room temperature (25°C), until further use.

### 2.5. Evaluation of Solid Nanoemulsifying Preconcentrate (SNP)

#### 2.5.1. Micromeritics and Reconstitution Properties

CUR-SNP (SP1 to SP3) was evaluated for powder properties, that is, tapped density, angle of repose, Carr's index, and Hausner's ratio. In addition, the drug loading efficiency, mean globule size distribution, and zeta potential were also determined. CUR-SNP (100 mg) was dispersed in 100 mL of phosphate buffer (pH 7.2) for 1 h using sonicator. After filtration, the filtrate was analyzed for *Z*-avg and zeta potential by photon correlation spectroscopy (PCS) Zetasizer (Nanosizer) Nano S90.

#### 2.5.2. *In Vitro* Dissolution Studies

Drug release was studied in a similar manner as described in Section 2.4.3.

#### 2.5.3. Surface Morphology

Surface morphology of CUR, Aerosil 200, and optimized CUR-SNP formulation (SP2) was examined using scanning electron microscope, SEM (EVO 18, Zeiss, Germany). Sample was fixed using double-sided adhesive tape to a brass specimen made electrically conductive by gold coating in vacuum [31]. Samples were imaged at different resolutions (2KX–12KX).

#### 2.5.4. Transmission Electron Microscopy (TEM)

Optimized CUR-SNP formulation (SP2) was investigated for globule shape and size using TEM. Samples (10 mg) were diluted with purified water, followed by gentle agitation. A drop of nanoemulsion was spread on a copper grid coated with carbon film. Thereafter, a drop of phosphotungstic acid (2% w/v) solution was carefully impinged on the copper grid. After exposure of one minute, excess solution was removed. Grid was air-dried at ambient temperature before loading in the microscope and analysed at 1,20,000x.

### 2.6. Solid State Characterization

#### 2.6.1. Differential Scanning Calorimetry

Thermal analysis of CUR, Aerosil 200, physical mixture of CUR with Aerosil 200, and CUR-SNP (SP2) were carried out using differential scanning calorimeter (DSC-204 F1, Netzsch-Gerätebau GmbH, Germany) under nitrogen purging (50 mL/min). Samples were placed in aluminium pans and heated from ambient temperature to 250°C at 10°C/min.

#### 2.6.2. X-Ray Powder Diffraction (XRPD)

Diffraction pattern of CUR, Aerosil 200, physical mixture of CUR and Aerosil 200 (1 : 1 ratio), and CUR-SNP (SP2) were obtained by XRPD (Bruker D8 Advance, Germany) to assess crystallinity. Scans were performed over 2*θ* range from 10 to 35°at 0.05 *θ*/sec step size/time.

#### 2.6.3. Stability Study

0.1 g of CUR-SNP formulation (SP2) and control (pure CUR) was weighed and dissolved in 100 mL of 0.01 mol/L alkali solution (pH 7.2), separately. Both solutions were stored in dark room and analysed using Raman spectroscopy after 5 h.

#### 2.6.4. *In Vivo* Animal Study

Overnight fasted three groups of guinea pigs, 250–300 g (*n* = 5), were fed with pure CUR loaded capsules (dose 100 mg/kg) and equivalent dose of optimized formulation (SP2) and control (water) via polyethylene tubing. The protocol (MMCP/IAEC/11/23) followed in the study was approved by the animal ethical committee of M. M. College of Pharmacy. Animals were kept at fasting during the study with free access to water. Guinea pigs were anesthetized using chloroform and blood samples (212 *μ*L) were withdrawn from the femoral vein in EDTA coated Eppendorf tubes at specified time intervals (0, 0.5, 1, 2, 4, 6, 8, and 10 h). Plasma samples were stored at −20°C till further analysis by HPLC.

## 3. Results

### 3.1. Solubility Studies

**Figure 1:**
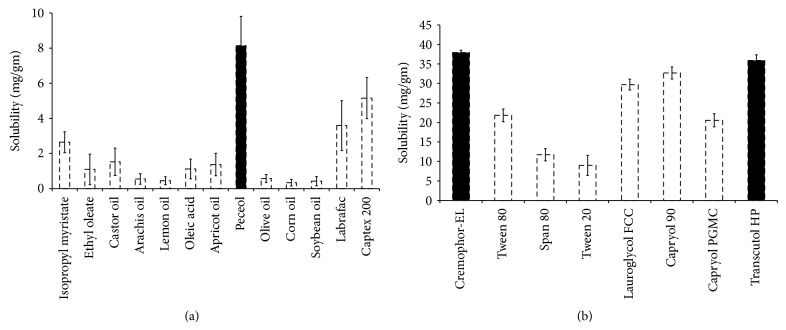
Solubility profile of CUR in different vehicles: (a) lipids, (b) surfactants and cosurfactants.

Solubility profile of CUR in different vehicles (oil, surfactant, and cosurfactant) is presented in Figure 1. Amongst the oils tested, Peceol showed the highest solubility (8.143 ± 0.671 mg/gm) for CUR and was thereby selected as the lipid phase (independent variable *X*_1_) for NP formulation (Figure 1(a)). Solubility of CUR was determined in various surfactants as shown in Figure 1(b). Cremophor-EL (C-EL), a nonionic surfactant with a medium length alkyl chain and an HLB value of 14, was selected (independent variable *X*_2_), on the basis of highest solubility of CUR (37.943 ± 0.592 mg/gm) in C-EL among the surfactants studied (Figure 1(b)). Similarly, T-HP, a medium chain fatty acid, was selected as the cosurfactant (independent variable *X*_3_) owing to good solubility (35.913 ± 0.415 mg/gm) of CUR and its compatibility with Cremophor-EL and Peceol.

### 3.2. Ternary Phase Diagram

**Figure 2:**
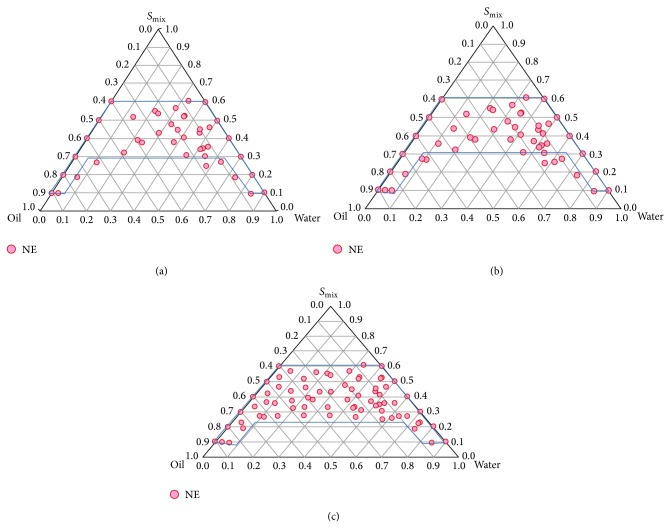
Schematic presentation of nanoemulsion region within the experimental domain (red dots) in the ternary phase diagram (values have been reduced to 1/100th in the plot): (a) *S*_mix_-1 : 2, (b) *S*_mix_-2 : 1, and (c) *S*_mix_-3 : 1.

Three variables, namely, oil, water, and mixture of surfactant and cosurfactant (*S*_mix_) (1 : 2, 2 : 1, and 3 : 1), were assessed on their impact on formulation variables (globule size, emulsification time, and *t*_85%_). Phase diagrams consisting of Peceol (oil), C-EL (surfactant), and T-HP (co-surfactant) were constructed (Figure 2). A total of 108 formulations were prepared using varying proportions of oil, surfactant, and cosurfactant. Region with red dots in the ternary diagram signifies stable self-emulsification region while the nonshaded ones show a monophasic region. Spontaneity of emulsification process was further enhanced by addition of cosurfactant, T-HP. Efficiency of emulsification was found to be good when surfactant/cosurfactant concentration was 50–55% w/w of CUR-SNP. CUR concentration between 8 and 13% w/w of the formulation was added to the boundary formulations as well as to the random points inside the emulsification area of the ternary phase diagrams. After identification of the nanoemulsion domain in the phase diagram, formulations were selected at desired component ratios of *S*_mix_ (3 : 1).

Box-Behnken design (BBD) was further applied to investigate the effect of independent variables oil, surfactant, and cosurfactant (*X*_1_, *X*_2_, and *X*_3_, resp.) on dependent variables, that is, globule size, emulsification time, and *t*_85%_ (*Y*_1_, *Y*_2_, and *Y*_3_, resp.). Analysis of variance (ANOVA) was applied to study the significance of regression, lack-of-fit test, correlation coefficient (*R*-square), and the adequate precision of quadratic model to estimate dependent variables (Table 3, Supplementary Data Table 1 in Supplementary Material available online at http://dx.doi.org/10.1155/2015/541510).

### 3.3. Evaluation of Liquid Nanoemulsifying Preconcentrate (LNP)

The model proposes the following equations for globule size, emulsification time, and *t*_85%_:

(2) Globule  size=+152.96+13.39X1−13.79X2+5.03X3+2.90X1X2−13.28X1X3−8.88X2X3+0.74X12+5.64X22−5.53X32,

where *F*-value 4.62, *R* ^2^ = 0.8559, and adequate precision = 8.450;

(3) Emulsification  time=+4.30−0.30X1+1.18X2−0.65X3+0.080X1X2+0.29X1X3+0.22X2X3−0.14X12−0.66X22+0.70X32,

where *F*-value 19.22, *R* ^2^ = 0.9611, and adequate precision = 16.160;

(4) t85%=23.63−0.60X1+2.02X2−0.97X3+0.42X1X2+0.085X1X3+0.50X2X3+0.94X12−0.82X22+0.23X32,

where *F*-value 4.19, *R* ^2^ = 0.8436, and adequate precision = 7.721.

A positive sign in the equation indicates a synergistic effect while a negative sign signifies antagonist effect, to the variable under consideration. Equation (2) reveals that significant factors affecting the response *Y*_1_ were the synergistic effects of *X*_1_, *X*_3_, *X*_1_ *X*_2_, *X*_1_^2^, and *X*_2_^2^ and antagonistic effects of *X*_2_, *X*_1_ *X*_3_, *X*_2_ *X*_3_, and *X*_3_^2^. In our study, globule size was increased in batch P2 at higher level of oil, low level of surfactant, and mid level of cosurfactant and decreased in batch P5 at low level of oil and cosurfactant and mid level of surfactant.

Y_2_ has the synergistic effects of *X*_2_, *X*_1_ *X*_2_, *X*_1_ *X*_3_, *X*_2_ *X*_3_, and *X*_3_^2^ and antagonistic effects of *X*_1_, *X*_3_, *X*_1_^2^, and *X*_2_^2^ (Equation (3)). Batch P10 shows higher emulsification time at mid level of oil, high level of surfactant, and low level of cosurfactant while batch P2 shows lower emulsification time at higer level of oil, low level of surfactant, and mid level of cosurfactant.

**Figure 3:**
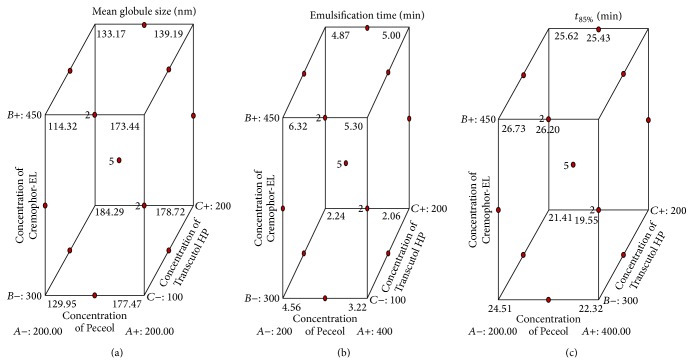
Cube surface graphs for the responses of Peceol, Cremophor-EL, and Transcutol HP.

*Y*_3_ has the synergistic effects of *X*_2_, *X*_1_ *X*_3_, *X*_2_ *X*_3_, *X*_3_^2^, and *X*_1_^2^ and antagonistic effects of *X*_1_, *X*_3_, and *X*_2_^2^ (Equation (4)). Batch P5 has maximum release rate at low level of oil and cosurfactant and mid level of surfactant and minimum release at mid level of oil, low level of surfactant, and high level of cosurfactant. These results are further supported by Figure 3.

Response *Y*_1_ (globule size) was significantly influenced by *X*_1_ and *X*_2_ (*P* < 0.01); *Y*_2_ (emulsification time) by *X*_1_, *X*_2_, *X*_3_, *X*_2_^2^, and *X*_3_^2^ (*P* < 0.01); and *Y*_3_ (*t*_85%_) by *X*_2_ (*P* < 0.001). ANOVA results reveal regression to be significant as per the quadratic model (Supplementary Data Table 2).

### 3.4. Identification and Evaluation of Optimum Formulation Using Desirability Function

**Figure 4:**
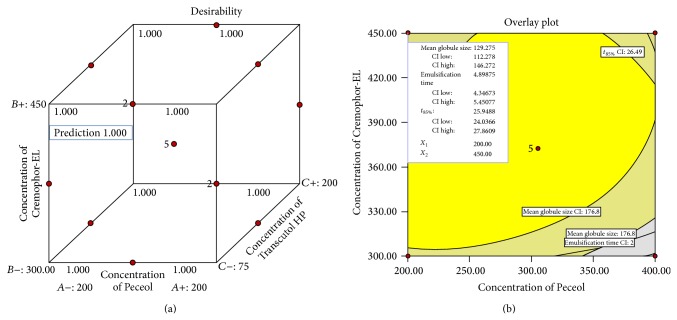
Cube surface and overlay plot for overall desirability [*D*] as a function of Peceol and Cremophor-EL.

Figure 4 shows the desirability and overlay plots in a variable range of oil and surfactant. Optimized levels of oil, surfactant, and cosurfactant were found to be 200 mg, 450 mg, and 150 mg, respectively. Formulations (P3, P5, and P12) have been suggested by software as optimized formulations (% bias <0.1%). Figure 4 shows the highest desirability factor (1.00), wherein a close agreement between the predicted and observed values was noticed.

### 3.5. Evaluation of Solid Nanoemulsifying Preconcentrate (SNP)

#### 3.5.1. Powder Flow Properties

Powder flow properties, optical clarity (absorbance at 425 nm), average particle size (*Z*_avg_), and percent drug loading (%DL) of the CUR-SNP are presented in Table 4. Formulations showed good flow characteristics with Carr's index (%) <20.0, Hausner's ratio <1.25, and angle of repose (*θ*) of 30.34°. Percent drug loading was reported in between 57.18 to 75.30% w/w, indicating high loading of CUR in the formulation without any significant loss during solidification. Zeta potential of CUR-SNP formulation (after dilution) varied from 7.12 to 9.89 mV signifying cationization of formed nanoemulsions. This indicates possibility of interaction between positively charged globules and negatively charged intestinal cells.

#### 3.5.2. *In Vitro* Dissolution Studies

**Figure 5:**
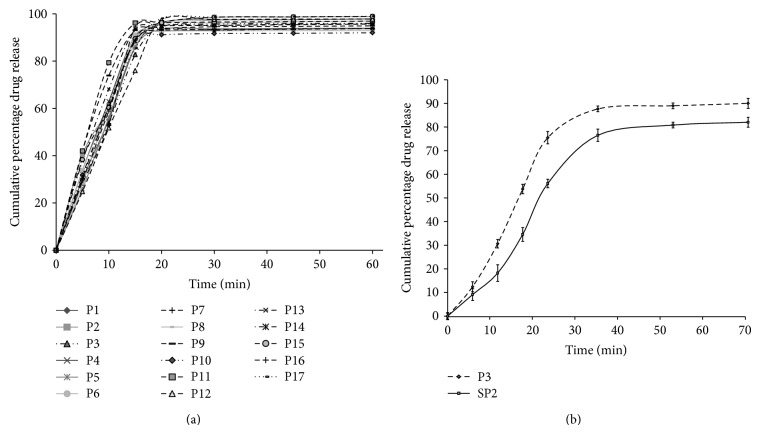
(a) Mean percent curcumin released for the optimal formulations (*n* = 3). (b) Mean percent curcumin released for the optimal formulations (P3 and SP2) (*n* = 3).

Formulations showed drug release of 2.611 ± 0.32% in initial 5 h, which represents sufficient resistance in the upper segment of the gastrointestinal tract.  Figure 5(a) illustrates drug release profile of CUR from LNP formulation (P1–P17) in phosphate buffer (pH 7.2). % drug release was immediate and more than 50% of the drug was released within initial 15 min (time was observed after dissolution of capsule shell). Comparative release profile of the optimized LNP (P3) and SNP (SP2) formulations in phosphate buffer (pH 7.2) is presented in Figure 5(b).

### 3.6. Solid State Characterization

#### 3.6.1. Differential Scanning Calorimetry (DSC)

DSC thermograms of CUR, Aerosil 200, physical mixture of CUR and Aerosil 200 (1 : 1 ratio), and optimized formulation CUR-SNP (SP2) are shown in Supplementary Data Figures 1(a)–1(d). A sharp endothermic peak of CUR appeared at 180.71°C showing its crystalline nature corresponding to CUR melting point. Aerosil 200 did not show any peak over the entire range of temperature. Similarly, CUR-SNP (SP2) did not show any melting endothermic peak, corresponding to its amorphous nature.

#### 3.6.2. X-Ray Powder Diffraction (XRPD)

XRPD of CUR describes its crystalline nature. Majority of peaks for CUR occurred at approximately 10°2*θ* angles, with highest intensity at 25.25°2*θ* (Supplementary Data Figure 2(a)). Aerosil 200 did not show any sharp diffraction peaks owing to its amorphous nature; however, halo pattern was observed with CUR loaded SNP formulation (Supplementary Data Figure 2).

#### 3.6.3. Scanning Electron Microscopy (SEM)

**Figure 6:**
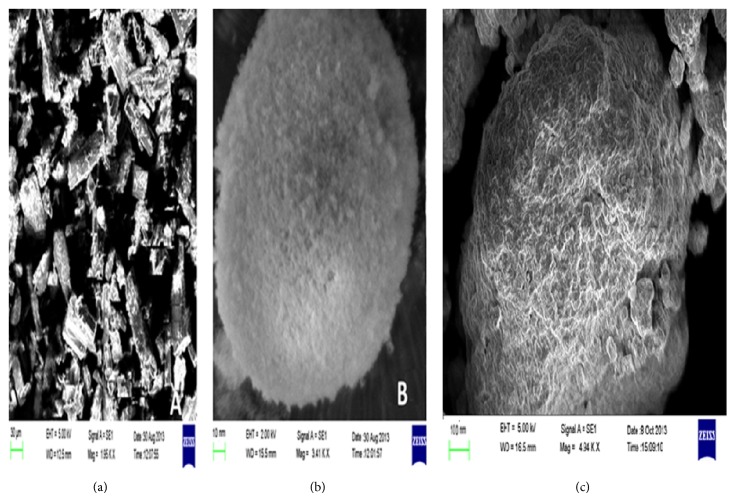
Scanning electron microscopy (SEM) images of CUR (a), Aerosil 200 (b), and optimized formulation (SP2) (c).

Surface morphology of CUR, Aerosil 200, and optimized formulation (SP2) was determined by SEM images in Figure 6. SEM images of CUR show the crystalline nature of drug indicating well defined edges (Figure 6(a)), while that of Aerosil 200 seems to be of porous amorphous nature (Figure 6(b)). Optimized SP2 formulation appeared to have a rough surface morphology, with CUR-LNP adsorbed on Aerosil 200 surface, as depicted in Figure 6(c) (SP2).

#### 3.6.4. Transmission Electron Microscopy

**Figure 7:**
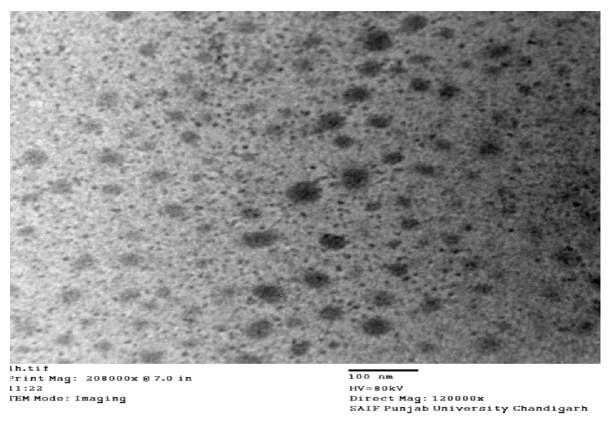
Transmission electron micrograph (TEM) image of optimized formulation (SP2).

Morphology of globules formed after dilution of SNP (SP2) was examined using transmission electron microscopy (Figure 7). Formation of spherical droplets with narrow droplet size (100 nm) indicated formation of nanoemulsions. Globules displayed no signs of coalescence, confirming the formation of a stable nanoemulsion.

#### 3.6.5. Stability Study

**Figure 8:**
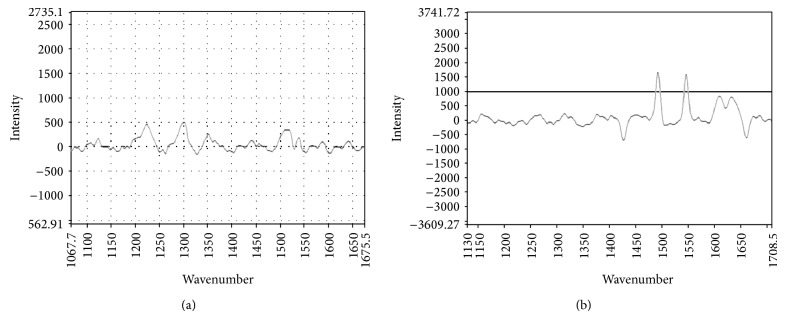
Raman spectrum of stability samples in alkali media (pH 7.2): control (Pure CUR) (a), SNP formulation (SP2) (b).

CUR specific peaks at 1360, 1470, 1510, 1601, and 1627 cm^−1^ were found in the solution containing formulation whereas no sign of CUR was observed in control group (Figure 8). Results ensure the protection of drug in alkaline media though stored for 5 h.

#### 3.6.6. *In Vivo* Animal Study

**Figure 9:**
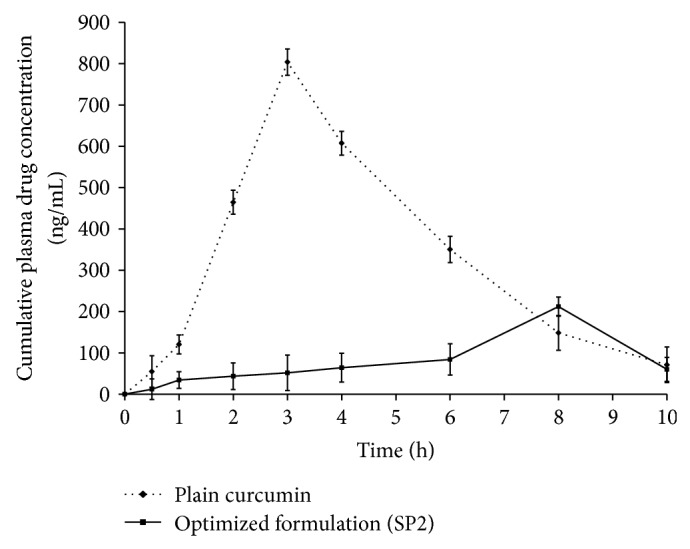
*In vivo* curcumin release from plain CUR and optimized SNP formulation (SP2) (*n* = 5).

Plasma drug concentration time profile of optimized formulation and plain CUR is presented in Figure 9. Insignificant plasma drug concentration (*C*_max⁡_ 212 ng/mL) was observed in group treated with optimized formulation (SP2) which may be due to limited absorption of formulation in colonic region.

## 4. Discussion

Current study is aimed at designing, developing, and optimizing nanoemulsifying preconcentrate formulation (NP) of curcumin (CUR). Three-factor, three-level design was run to evaluate the independent formulation variables (quantity that affects the response) which include amounts of Peceol (oil), Cremophor-EL (surfactant), and Transcutol HP (cosurfactant) (*X*_1_, *X*_2_, *X*_3_, resp.). Dependent variables (measured response that is the subject of study) include mean globule size in phosphate buffer at pH 7.2 (*Y*_1_), emulsification time (*Y*_2_), and *t*_85%_ (*Y*_3_).

Solubility study was performed to evaluate the suitability of excipients for CUR NP formulations that could solubilize CUR but at the same time avoid its precipitation upon dilution in the gut lumen* in vivo* [32]. Based on solubility data, Peceol was opted as an excipient of choice as it showed the highest solubility of CUR (8.143 ± 0.671 mg/gm). Besides the highest solubility of CUR, Peceol further showed favourable emulsification efficacy with other ingredients and was therefore selected as a lipid phase for CUR NP formulation. Nonionic surfactants are generally considered for oral administration because of being safer than the ionic surfactants [33, 34]. Additionally, they can produce reversible changes in intestinal mucosa, thus leading to enhancing drug permeability [35]. Therefore, Cremophor-EL was selected as the surfactant which also shows good emulsification with T-HP.

Ternary phase diagrams (Figure 2) were prepared to locate the nanoemulsion region and optimize the ranges of independent variables (oil, surfactant, and cosurfactant). Addition of surfactant was limited to avoid instability of nanoemulsions caused by poor localisation of surfactant at oil water interface [36]. C-EL (as surfactant) solubilized only a small fraction of CUR; hence cosurfactant, Transcutol HP (T-HP), was added to the formulation. Highest nanoemulsion region was observed with *S*_mix_ ratio of 3 : 1. Upon increasing the concentration of surfactant to cosurfactant up to 4 : 1, reduction in the nanoemulsion region was noted. Therefore, it was concluded that *S*_mix_ should not be used in a ratio greater than 3 : 1. Further, phase diagram indicates formation of w/o type nanoemulsion when oil: *S*_mix_ was 1 : 8 upon addition of 10% of water. However, further increase in water concentration resulted in formation of o/w type nanoemulsion. This suggested that the present formulation is dynamic and can be used to prepare o/w as well as w/o type nanoemulsions.

Box-Behnken design of experiment has an advantage over other designs that it does not contain combinations for which all factors are simultaneously at their highest or lowest levels. Hence, this design is beneficial in the sense that experimentation under extreme conditions for which generally unsatisfactory results are obtained may not be performed [37].  Figure 3(a) shows that increased globule size was recorded on increasing oil concentration while decreasing the concentration of surfactant and cosurfactant. Results suggested an inverse relationship between the mean droplet size and surfactant concentration (Supplementary File Figure 3). Such behaviour can be described by the fact that stabilization of the oil droplets is a result of the localization of the surfactant molecules at the oil water interface [38].

Emulsification time is considered to be an important parameter while describing the self-emulsifying ability of a lipoidal formulation [39].  Figure 3(b) reveals that emulsification time increased at higher level of C-EL. Higher viscosity of C-EL can be attributed to such observation, resulting in a slow rate of emulsification [40]. On the other hand, higher lipid concentration may increase the interfacial fluidity and accelerate the progress of emulsification process, resulting in lesser emulsification time [41, 42]. Therefore, it is concluded that higher levels of oil and lower levels of surfactant can be used for an optimized formulation with minimum emulsification time.

Figure 3(c) shows that higher surfactant concentration increases the time to 85% drug release, possibly due to formation of viscous crystalline gel at the interface. This relationship agrees with the results of Trotta, 1999, who proposed phase transformation from one liquid crystalline structure to another during the emulsification process [43]. Furthermore, synergistic effect of cosurfactant with oil, to decrease the *t*_85%_, has been represented by Figure 3(c). Therefore, it was concluded that, to reduce the *t*_85%_, lower levels of surfactant and high levels of oil and cosurfactant are required in an optimized formulation.

SEM micrographs of surface-adsorbed CUR-LNP were similar to that of Aerosil 200 indicating that the LNP is adsorbed on the surface of fused silica, as depicted in Figure 6(c) (SP2). Release rate is strongly influenced by surface morphology of the particles.* In vitro* drug release profile of encapsulated SNP formulation showed a lower drug release compared to LNP, probably due to the porous surface of the particles. Formulation was observed to exhibit gastric resistance during first 5 h in simulated gastric fluid and thereafter exhibited an immediate release (*t*_85%_ < 30 min) in phosphate buffer (pH 7.2).

Positively charged globules have been reported to have more interaction with the mucosal membrane of GIT than intestinal cells which carry negative charges because of the presence of mucosal fluid [38]. In order to make a high-energy barrier against coalescence of the dispersed droplets, high values of absolute zeta potential should preferably be achieved [44]. Physical stability of nanoemulsion was further supported by transmission electron microscopy (TEM), (Figure 7) where the individual globules were found to be nonaggregated to each other. When particle sizing data observed with dynamic light scattering (DLS) technique was compared to TEM images, the aggregation state of the particles can be determined. It has been observed that DLS measured diameter was slightly larger than the TEM size, which suggests unagglomeration of globules. Results were further supported by observed polydispersity index (<0.5), which shows that globules may be big but are nonaggregated to each other.

DSC thermograms indicated a change in physical state of the drug from crystalline to amorphous state when formulated as the NP formulation. Moreover, compatibility of CUR with the excipients was confirmed, owing to absence of any additional peaks in the DSC traces of optimized formulation. Amorphous state of CUR was further confirmed by the presence of a halo pattern in PXRD (Supplementary Data Figures 1(c) and 1(d)), indicating complete solubilization of CUR and resulting amorphization in lipoidal formulation.

Plasma drug concentration time profile signifies insignificant amount of drug in plasma (*C*_max⁡_ = 212 ng/mL), which may lead to either degradation of drug or localised delivery to targeted (colonic) site. Stability study indicated that the formulation remains stable in alkaline conditions (pH 7.2) even after being kept for 5 h. Therefore, it may confer that limited systemic absorption (plasma drug profile) and targeting the intact drug in the large intestine (*in vitro* release) favour the conditions required for localised delivery [45]. Limited systemic absorption of drug was probably due to limited colonic mucosal surface area compared to small intestine. Therefore, it could be concluded that the optimized formulation can be successfully used for localised delivery in colonic region.

## 5. Conclusion

In the present study, liquid and solid nanoemulsifying preconcentrate formulation of curcumin was developed using the design of experiment methodology. Peceol (200 mg), Cremophor-EL (450 mg), and Transcutol HP (150 mg) were selected to formulate curcumin nanoemulsifying preconcentrate formulation. Results suggested that globule size was significantly influenced by increasing the concentration of oil (Peceol). In contrast, higher levels of Peceol and lower levels of Cremophor-EL led to lower emulsification time. Plasma drug profile signifies localised delivery of drug at colonic sites. The results confer the suitability of selected curcumin loaded nanoemulsifying preconcentrate formulation in the treatment of colon cancer.

## Supplementary Material

Supplementary Tables 1-2 presents the ANOVA analysis of the model equation.

## Figures and Tables

**Table 1:** Data of analytical method validation of CUR by HPLC.

Parameters	Results	Acceptance criteria
Linearity (*R* ^2^) (10–100 *μ*g/mL)	0.9995	>0.999
Accuracy (% Mean ± SD)	99.12 ± 1.60	98–102
Robustness (% RSD)	1.12	<2
Repeatability precision (% RSD)	0.37	<1
Intermediate precision (% RSD)	0.58	<2
LOD (*μ*g/mL)	0.1	Signal : Noise ratio should be 3 : 1

**Table 2:** Composition of nanoemulsifying preconcentrate formulation using Box-Behnken design.

	Levels					
Independent factors	Low		Middle		High	
	Code	Actual (mg)	Code	Actual (mg)	Code	Actual (mg)
*X*_1_: Conc. of Peceol (mg)	−1	200	0	300	1	400
*X*_2_: Conc. of Cremophor EL (mg)	−1	300	0	400	1	450
*X*_3_: Conc. of Transcutol HP (mg)	−1	100	0	150	1	200

Constraints						
Dependent variables			Goal			
*Y*_1_: Mean globule size (nm)			Minimize			
*Y*_2_: Emulsification time (min)			In range			
*Y*_3_: time required for drug release (85%); *t*_85%_ (min)			In range			

**Table 3:** Combination levels of independent variables and the outcome of response variables by Box-Behnken design.

Batch No.	Independent factor			Expected response			Predicted response		
	*X*_1_^@^	*X*_2_^$^	*X*_3_^*^	*Y*_1_^≈^	*Y*_2_^*ν*^	*Y*_3_^*β*^	*Y*_1_^≈^	*Y*_2_^*ν*^	*Y*_3_^*β*^
P1	−1	−1	0	166.81	2.60	23.75	162.65	2.71	22.74
P2	+1	−1	0	176.83	2.00	21.36	183.63	1.94	20.71
P3	−1	+1	0	136.14	4.84	25.30	129.28	4.90	25.95
P4	+1	+1	0	157.72	4.56	24.58	161.85	4.45	25.59
P5	−1	0	−1	105.21	6.00	26.14	116.49	6.11	26.45
P6	+1	0	−1	169.50	4.65	25.14	169.81	4.93	25.09
P7	−1	0	+1	153.44	4.50	24.29	153.09	4.22	24.34
P8	+1	0	+1	164.62	4.31	23.63	153.31	4.20	23.32
P9	0	+1	+1	160.12	4.25	21.78	152.96	4.03	22.48
P10	0	+1	−1	147.60	6.12	26.49	143.14	5.95	25.53
P11	0	−1	+1	176.31	2.12	18.59	180.76	2.29	19.55
P12	0	−1	−1	128.30	4.86	25.29	135.44	5.08	24.59
P13	0	0	0	156.15	4.12	22.93	152.96	4.30	23.63
P14	0	0	0	145.90	4.21	23.95	152.96	4.30	23.63
P15	0	0	0	160.10	4.31	24.05	152.96	4.30	23.63
P16	0	0	0	158.81	4.85	24.81	152.96	4.30	23.63
P17	0	0	0	143.92	4.00	22.43	152.96	4.30	23.63

^*^Standard deviation of observed responses found within ± 5%.

^@^Peceol (oil); ^$^Cremophor EL (polymer); ^*^Transcutol HP (adsorbent).

^≈^Mean globule size (nm); ^*ν*^Emulsification time (min);  ^*β*^ *t*_85%_ (min).

**Table:** (a) Optimized CUR-LNP

Code	Globule size		Drug Release	Viscosity (Poise)	Drug loading (%)
	*Z*-avg. (nm)	PDI	*t*_85%_ (min)		
P3	**136.1**	**0.498**	**25.30**	**4.98**	**70.13**
P5	105.2	0.690	26.14	4.12	64.21
P12	128.3	0.323	25.29	5.57	61.86

**Table:** (b) Optimized CUR-SNP

Code	*Z*-avg (nm)	PDI	*t*_85%_ (min)	Powder properties			Drug Loading (%)
				Carr's Index (%)	Hausner's ratio	Angle of Repose (*θ*)	
SP1^*δ*^	141.8	0.428	26.17	20.16	1.29	34.43	57.18
SP2^◊^	**153.3**	**0.510**	**28.89**	**18.64**	**1.18**	**32.64**	**75.30**
SP3^×^	169.1	0.628	31.12	17.64	1.16	31.12	69.15

^*δ*,◊,×^Nanoemulsion preconcentrate with LNP: Aerosil 200; 1 : 1, 1 : 5, 1 : 10 respectively.
