# Supplementary material for: Development and Evaluation of Nanoemulsifying Preconcentrate of Curcumin for Colon Delivery
Source: ScientificWorldJournal. 2015 Mar 11;2015:541510. doi: 10.1155/2015/541510 (PMC4377495; doi:10.1155/2015/541510)
Supplement: Supplementary file 1 — Supplementary Tables 1-2 presents the ANOVA analysis of the model equation. [file 541510.f1.pdf]

Supplementary File

Table 1: Summary of each factor effect and its p-values for responses (Mean Globule size, emulsification time and t85%)

Factor	$Y_1^{\approx}$		Y_2^{ν}		Y_3^{β}	
	Factor effect	p value	Factor effect	p value	Factor effect	p value
Intercept	152.96		4.30		23.63	
$X_1^{@}$	13.39	0.0081	-0.30	0.0385	-0.60	0.1911
$X_2^{\$}$	-13.79	0.0070	1.18	<0.0001	2.02	0.0017
X_3^{*}	5.03	0.2124	-0.65	0.0009	-0.97	0.0510
X_1X_2	2.90	0.5930	0.080	0.6489	0.42	0.4969
X_1X_3	-13.28	0.0374	0.29	0.1284	0.085	0.8881
X_2X_3	-8.88	0.1304	0.22	0.2371	0.50	0.4215
X_1^2	0.74	0.8868	0.14	0.4366	0.94	0.1426
X_2^2	5.64	0.3004	-0.66	0.0049	-0.82	0.1899
X_3^2	-5.53	0.3096	0.70	0.0036	0.23	0.7001

$^{@}$Peceol (oil); $^{\$}$Cremophor EL (polymer); * Transcutol HP (adsorbent)
$^{\approx}$Mean globule size (nm); $^{\nu}$Emulsification time (min); $^{\beta}$ t_{85}% (min)

Table 2: ANOVA summary of measured response (Mean globule size, Emulsification time and $t_{85\%}$)

	Model							Lack of Fit	
**Response**	**F Value**	**F-Significance**	**R^2**	**Adjusted R^2**	**S.D.**	**% CV**	**Adequate Precision**	**F value**	**F significance**
**$Y_1^{≈}$**	**4.62**	**0.0280**	**0.855**	**0.670**	**10.36**	**6.75**	**8.450**	**3.08**	**0.152**
**Y_2^{v}**	**19.22**	**0.0004**	**0.961**	**0.911**	**0.34**	**7.91**	**16.160**	**1.11**	**0.444**
**Y_3^{β}**	**4.19**	**0.0360**	**0.843**	**0.642**	**1.17**	**4.90**	**7.721**	**2.19**	**0.232**

$^{≈}$Mean globule size (nm); ^{v}Emulsification time (min); $^{\beta}$ t85% (min)

Figure 1. DSC scans of Curcumin [A]; Aerosil® 200 [B]; CUR - Aerosil® 200 Physical mixture [C]; and optimized formulation, SP2 [D]

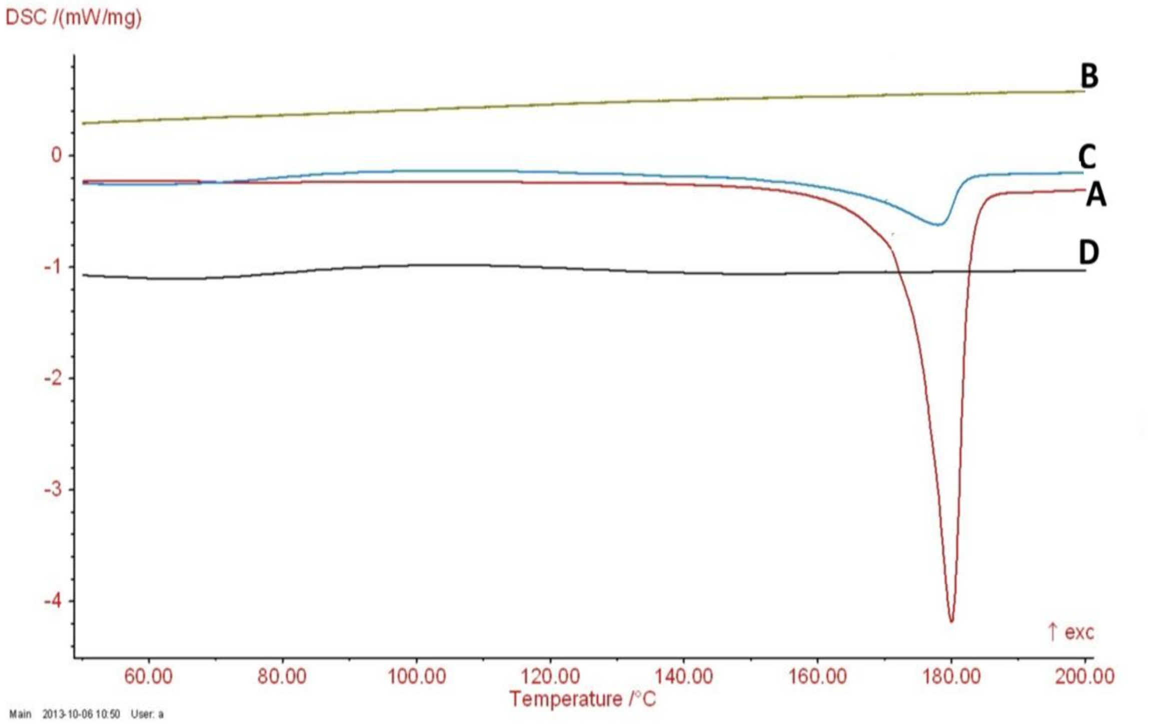

Figure 2. Overlay of XRD pattern of free Curcumin [A]; Aerosil® 200 [B]; CUR - Aerosil® 200 Physical mixture [C]; and optimized formulation, SP2 [D]

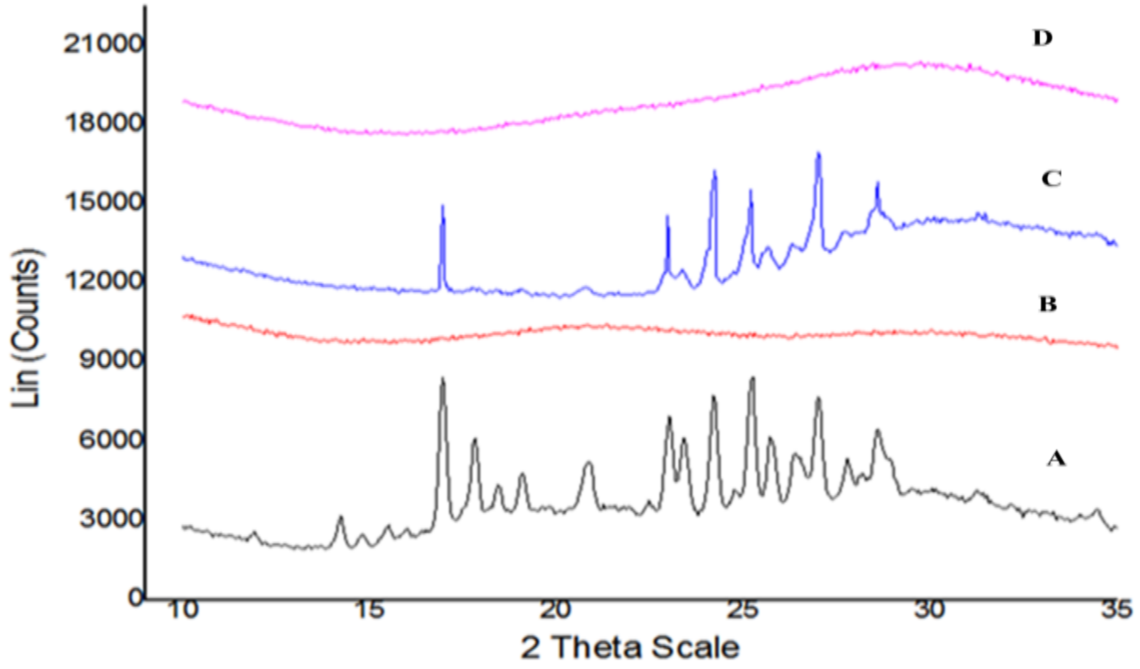

Figure 3. Evaluation of the standardized effects of the Peceol (X_1), Cremophor® EL (X_2) and Transcutol® HP (X_3), and their interactions on mean globule size (Y_1), Emulsification time (Y_2), time taken to release 85% drug (t85%) (Y_3)

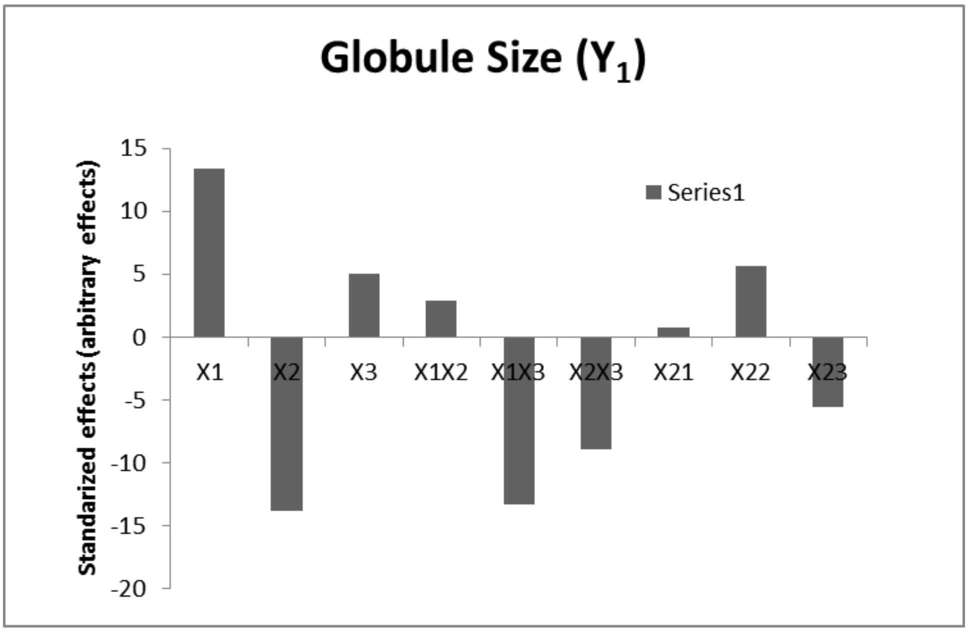

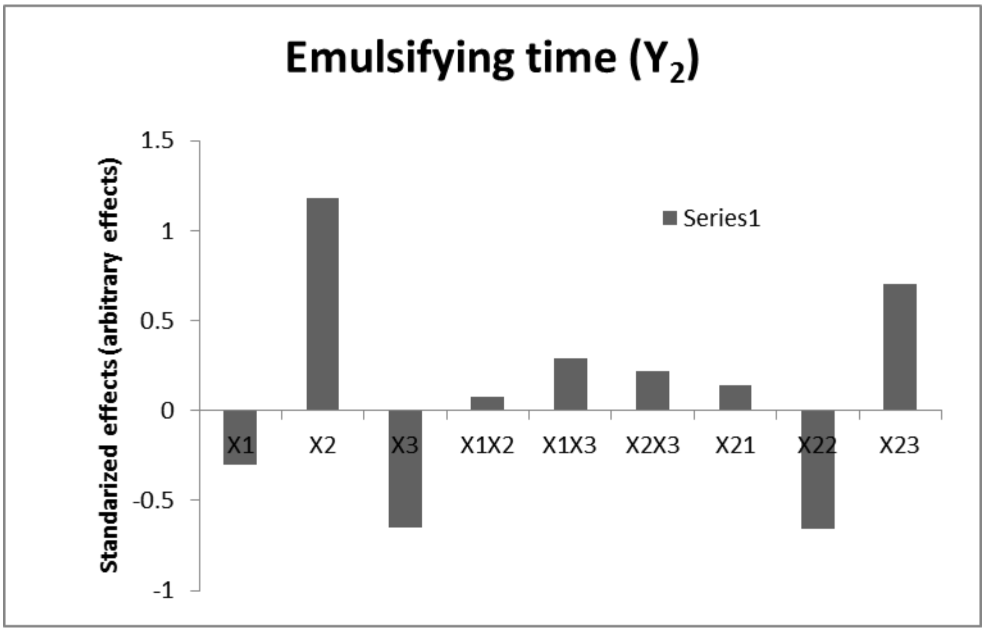

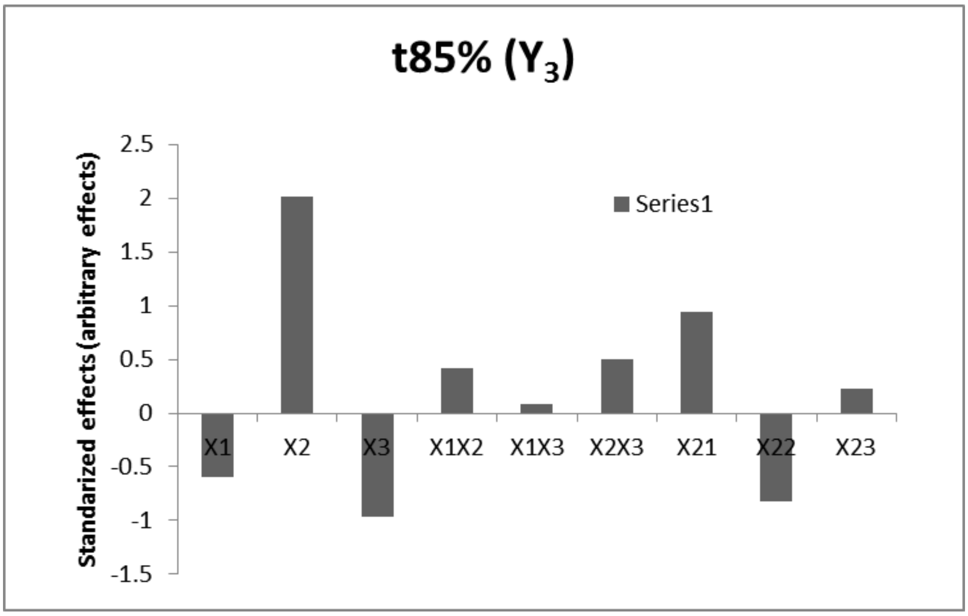
t85% (Y_3)
Standarized effects (arbitrary effects)
2.5
2
1.5
1
0.5
0
-0.5
-1
-1.5
Series1
X1
X2
X3
X1X2
X1X3
X2X3
X21
X22
X23
